# Bridging Offline Experience and Digital Commerce: How Tourism-Derived Information Reduces Uncertainty and Shapes Purchase Intention in Cross-Border E-Commerce

**DOI:** 10.3390/bs16071042

**Published:** 2026-06-23

**Authors:** Sangyoon Jang, Li Cai, Sukjae Park, Zuankuo Liu

**Affiliations:** 1 School of Business, Shandong Xiehe University, Jinan 250109, China; jangsangyoon@sdxiehe.edu.cn; 2 Business School, Shandong Normal University, Jinan 250358, China; 3 Business School, Woosuk University, Wanjugun 55338, Republic of Korea

**Keywords:** cross-border e-commerce, tourism-derived information, purchase intention, product uncertainty, product familiarity, perceived diagnosticity

## Abstract

Cross-border e-commerce (CBEC) has emerged as a critical mode of international trade; however, product uncertainty and transaction risk remain persistent barriers to purchase decisions. While digital platforms have developed various solutions, the role of offline experiential knowledge in shaping online purchase behavior remains underexplored. This study examines how tourism-derived information influences purchase intention in CBEC. Drawing on transaction cost theory and uncertainty reduction theory, we propose that tourism-derived information enhances product familiarity and perceived diagnosticity, which subsequently reduce product uncertainty and increase cross-border purchase intention, and further examine the moderating role of transaction uncertainty. A four-week survey in March 2026 collected data from 325 Chinese consumers who had visited Korea and encountered Korean cosmetics and beauty products; data were analyzed using PLS-SEM. Results show that tourism-derived information significantly enhances product familiarity and perceived diagnosticity while directly reducing product uncertainty; reduced product uncertainty, in turn, positively influences purchase intention. Transaction uncertainty strengthens the negative effect of product uncertainty on purchase intention. By reconceptualizing tourism experience as an experience-based informational resource in CBEC and providing a multidimensional perspective on consumer uncertainty, this study contributes to consumer behavior research in digital commerce and offers practical insights for CBEC platform operators and cross-border retailers.

## 1. Introduction

Advances in digital technology and online platforms have rapidly transformed global consumption and distribution patterns, making cross-border e-commerce (CBEC) an increasingly important mode of international trade (Kim et al., 2017). By facilitating direct connections between overseas sellers and consumers via online platforms, CBEC has substantially reduced the spatial and institutional barriers traditionally associated with international trade (Cassia & Magno, 2022; Kim et al., 2017). This enables consumers to access a broader range of products at competitive prices, while simultaneously enhancing firms’ opportunities to penetrate overseas markets (Huang & Chang, 2019; Mou et al., 2020).

However, compared with domestic e-commerce, CBEC is inherently associated with a heightened level of uncertainty (Ma et al., 2025; Mou et al., 2020). Consumers are required to make purchasing decisions without being able to directly inspect products (Pavlou et al., 2007), while simultaneously facing transaction-related risks, such as international delivery, customs clearance, unforeseen additional costs, language barriers, and concerns regarding the protection of personal information (X. Chen & Kim, 2021; Huang & Chang, 2019; Mou et al., 2020). From the perspective of transaction cost theory, this uncertainty elevates the transaction costs that consumers must bear and consequently reduces purchase intention (Lee & Xiong, 2026; Williamson, 2010).

To address these challenges, digital commerce platforms have implemented various technological solutions, such as virtual reality (VR) and augmented reality (AR), to assist consumers in evaluating products and mitigating uncertainty (Sun et al., 2022; Wedel et al., 2020). However, despite these technological advancements, product uncertainty and transaction risk continue to represent significant barriers to CBEC purchase decisions. One possible reason is that digitally mediated cues are inherently constrained by screen-based interfaces and filtered distribution channels (Weathers et al., 2007). In contrast, tourism experiences allow consumers to directly observe, compare, and evaluate products within their country-of-origin market context, thereby providing multisensory and context-rich information that digital solutions cannot fully replicate (Yim et al., 2007). This suggests that uncertainty reduction in CBEC may depend not only on digital technologies but also on consumers’ access to credible and experience-based information sources.

According to uncertainty reduction theory, tourism experiences can function as significant information sources because they offer a comprehensive informational environment in which consumers can directly observe and interact with a specific country’s products and retail environments. Given that shopping constitutes a fundamental aspect of this experience (Jin et al., 2017; Moscardo, 2004), tourists acquire knowledge and form impressions of local products through their exposure to those products at the destination (De Nisco et al., 2017; Elliot et al., 2011). Such experiences may subsequently provide experience-based cues that consumers utilize when evaluating and purchasing products from a country (Mainolfi & Marino, 2020). Thus, tourism-derived information may function as a unique experience-based informational resource that shapes consumers’ product knowledge and evaluation criteria in post-visit purchase contexts.

Previous studies have identified several mechanisms through which tourism promotes international trade. Specifically, tourism stimulates post-visit import demand by fostering familiarity with products from the destination country (Brau & Maria Pinna, 2013), facilitates trade through interpersonal and business networks established during travel (El-Sahli, 2018), and disseminates information about products and markets, thereby reducing search costs for buyers and sellers (Santana-Gallego et al., 2016). However, these studies have primarily examined the tourism–trade relationship at the macro-level using national-level data. Consequently, they provide limited insight into how tourism-derived information influences consumer decision-making at the individual level. In particular, little is known about how tourism-derived information reduces product uncertainty and subsequently shapes purchase intention in digital commerce environments such as CBEC. To address this gap, this study conceptualizes tourism-derived information as an experience-based informational resource that influences post-visit consumer decision-making. Unlike prior CBEC studies that have primarily focused on platform characteristics, trust mechanisms, and technological solutions to reduce uncertainty (Huang & Chang, 2019; Mou et al., 2020), this study examines how information acquired through tourism functions as an uncertainty-reducing resource in online purchase contexts.

This study focuses on the micro-level mechanisms through which tourism-derived information influences purchase intention in the CBEC context. The effectiveness of information in reducing uncertainty depends not only on the amount of information acquired but also on the extent to which that information is perceived as useful for product evaluation (Keller & Staelin, 1987). During tourism experiences, consumers are repeatedly exposed to products through local retail stores and various distribution channels, thereby accumulating product-related knowledge and developing a sense of familiarity with those products (Baker et al., 1986; Johnson & Russo, 1984). Furthermore, because information acquired in real-world market environments provides richer and more contextualized cues than information obtained through digital channels, consumers are more likely to perceive such information as diagnostic for evaluating product quality and performance (Kempf & Smith, 1998). Unlike trust and brand image, which reflect consumers’ overall impressions of sellers or brands (Pavlou et al., 2007), product familiarity and perceived diagnosticity more directly capture how consumers process and utilize product-related information. As such, these constructs are particularly well-suited for capturing the cognitive role of tourism-derived information in consumer decision-making. Thus, this study pays particular attention to the role of product familiarity and perceived diagnosticity as key cognitive mechanisms through which tourism-derived information reduces product uncertainty.

Furthermore, this study explores the potential variability in the process by which product uncertainty influences purchase intention, contingent upon transaction uncertainty. In the CBEC context, various risks may emerge during the transaction execution phase, including international delivery, customs procedures, additional costs, personal information protection, and communication with sellers (Y. Guo et al., 2018; Lee & Xiong, 2026; Mou et al., 2020). Such transaction uncertainty may constrain consumers’ purchase decisions, independently of their evaluation of the product itself. In other words, even when consumers perceive relatively low uncertainty regarding a foreign product, their actual cross-border purchase intention may still weaken if they perceive high uncertainty regarding the overall transaction process.

Against this background, this study addresses prior research gaps by developing and testing a micro-level framework that links tourism experience to consumer purchase behavior in the CBEC context. Drawing on transaction cost theory and uncertainty reduction theory, this study proposes that tourism-derived information functions as a critical cognitive resource that enhances product familiarity and perceived diagnosticity, thereby reducing product uncertainty and increasing cross-border purchase intention. Furthermore, this study examines the boundary condition of this process by investigating whether perceived CBEC transaction uncertainty moderates the relationship between product uncertainty and purchase intention, thereby shaping the extent to which reduced product uncertainty translates into actual purchase intention. This study contributes to the growing body of research on consumer behavior in digital commerce by elucidating how offline experiential information formed through tourism shapes online purchase decision-making in CBEC.

## 2. Literature Review

### 2.1. Transaction Cost Theory: Uncertainty in CBEC

According to transaction cost theory, a transaction is not merely a price exchange but a process that involves various costs, such as search, bargaining, monitoring, and enforcement (Williamson, 2010). From this perspective, uncertainty emerges as a pivotal factor that elevates transaction costs; the greater the difficulty for transacting parties to anticipate the behavior of the other party or the transaction’s outcome, the higher the costs incurred to execute the transaction (Liang & Huang, 1998). In the CBEC context, a variety of stakeholders—including consumers, sellers, e-commerce platforms, payment service providers, and international logistics service providers—engage in interactions with one another (Kim et al., 2017). Due to the transnational nature of these transactions, the process is inherently more prolonged and complex compared to domestic e-commerce (Y. Guo et al., 2018). Consequently, consumers encounter increased levels of uncertainty throughout the transaction process in the CBEC environment (Mou et al., 2020).

A review of previous research on general online shopping and CBEC suggests that, in the CBEC context, consumer-perceived uncertainty can be categorized into two primary dimensions based on the nature of the uncertainty. The first dimension is product uncertainty, which emerges during the product evaluation phase. Product uncertainty is characterized by consumers’ difficulty in accurately assessing, in advance, the quality, performance, or suitability of a product for their needs (Dimoka et al., 2012; Hong & Pavlou, 2014). In the online environment, consumers are unable to physically inspect or experience products, resulting in limited direct cues for product evaluation (Dimoka et al., 2012; Featherman & Pavlou, 2003; Pavlou et al., 2007). This issue may be exacerbated in the CBEC context, where information asymmetry is more pronounced (Lee & Xiong, 2026; Mou et al., 2020). Although CBEC enables consumers to compare a broader array of foreign brands and similar products than domestic e-commerce, it also increases the volume of information consumers must process and the complexity of product comparisons (Lee & Xiong, 2026). Additionally, unfamiliar product descriptions or website displays may further complicate consumers’ ability to accurately interpret product information and assess product quality (Huang & Chang, 2019). While online reviews and ratings may serve as indicators to mitigate information asymmetry (L. Zhu et al., 2020), concerns about review manipulation and credibility persist (Román et al., 2023). Moreover, in the CBEC environment, language barriers, along with cultural and institutional differences, may amplify the risk of information misinterpretation, thereby further impairing consumers’ ability to accurately evaluate product quality or suitability (Mou et al., 2020). Therefore, product uncertainty in CBEC can be conceptualized as a fundamental form of uncertainty stemming from the necessity for consumers to evaluate product attributes based on limited and imperfect information.

The second dimension is transaction uncertainty, which emerges during the transaction execution phase. Transaction uncertainty refers to consumers’ overall perception of whether a transaction can be completed without problems, apart from the attributes of the product itself. CBEC involves transactions that are inherently more complex and subject to greater external environmental constraints than domestic transactions (Huang & Chang, 2019; Lee & Xiong, 2026; W. Zhu et al., 2019). Previous research has indicated that consumers may encounter various concerns at this stage of the transaction. For instance, W. Zhu et al. (2019) posited that consumers might face risks associated with delivery, financial transactions, and time during the CBEC transaction process. X. Chen and Kim (2021) identified challenges such as foreign-language difficulties, logistical infrastructure barriers, payment infrastructure barriers, and high import tariffs as significant constraints on CBEC transactions. Mou et al. (2020) focused on post-contractual uncertainty arising in the post-purchase phase, highlighting delivery risk, privacy risk, financial risk, and confiscation risk as critical concerns. Furthermore, Huang and Chang (2019) noted that in the CBEC context, transaction costs include communication costs due to language barriers, waiting costs from international shipping, and return costs stemming from complex return procedures. Taken together, these studies suggest that during the transaction execution process in CBEC, consumers are likely to be apprehensive about various issues, such as delays or errors in international delivery, unexpected additional costs, customs-related challenges, the protection of personal and payment information, communication difficulties with sellers, and complications in returning products.

To address these challenges, prior research in the CBEC context has proposed a variety of uncertainty-reducing mechanisms. One stream focuses on trust-based mechanisms, such as platform quality, seller reputation, institutional guarantees, and payment security (K. Liu et al., 2025; Ma et al., 2025; Wang et al., 2023; W. Zhu et al., 2019). Another stream focuses on information quality and social information cues, including online reviews, ratings, electronic word-of-mouth (e-WOM), and the accuracy and completeness of product information, as means of reducing consumer uncertainty (Huang & Chang, 2019; Lee & Xiong, 2026; Zhou et al., 2025). A third stream explores how emerging digital technologies, such as live-streaming commerce, augmented reality (AR), and AI-driven recommendations, can reduce product uncertainty by providing richer, more vivid, and more interactive product information (D. Liu & Yu, 2024; Lu & Chen, 2021; Sun et al., 2022; Xu et al., 2024). However, these approaches share a common reliance on platform-mediated or digitally generated information and may not sufficiently address the fundamental information deficiencies consumers face when evaluating cross-border products. Consequently, there remains a need to explore alternative information sources beyond digital platforms that can help consumers evaluate foreign products more effectively.

### 2.2. Uncertainty Reduction Theory: Tourism-Derived Information

Uncertainty reduction theory (URT) asserts that individuals actively gather information to diminish uncertainty and enhance the predictability of outcomes when interacting with unfamiliar objects (Berger & Calabrese, 1974). Although initially conceptualized within the context of face-to-face interactions, the theory has been extended to encompass various digital environments, such as online shopping and social media. In these contexts, URT serves as a pivotal theoretical framework for understanding how individuals employ diverse information sources to mitigate uncertainty (Antheunis et al., 2010; Hwang & Youn, 2023; Yan & Gong, 2023). According to URT, individuals enhance their understanding of a target’s characteristics and quality through information-seeking behaviors, thereby enabling more stable and confident evaluation (Berger & Calabrese, 1974).

URT classifies information acquisition strategies into passive, active, and interactive strategies (Hwang & Youn, 2023; Yan & Gong, 2023). Passive information acquisition involves the collection of information through observation without engaging directly with the subject. In the tourism context, this includes observing product displays, pricing details, advertising, store atmosphere, and the purchasing behavior of local consumers in retail environments. Active information acquisition refers to seeking information through third parties without direct interaction with the subject. Tourists may seek recommendations from local residents or companions regarding specific products or conduct online searches to obtain additional information about products encountered during their visit. Finally, interactive information acquisition refers to obtaining information through direct interaction with the subject or sellers. For instance, tourists may test products in stores or engage with sales personnel to assess product quality and determine the suitability of the products for their needs.

From this perspective, tourism offers a distinctive, integrated experiential environment wherein all three information acquisition strategies operate concurrently. Generally, when consumers encounter foreign products within their domestic markets, they tend to depend on limited and filtered information provided through local distribution channels (Ortega-Egea & García-de-Frutos, 2021; Yim et al., 2007). Conversely, in the context of tourism, consumers have the opportunity to directly observe and experience products within their country-of-origin market (Yim et al., 2007), thereby enabling them to form impressions and attitudes toward these products (Mainolfi & Marino, 2020). By visiting various retail channels, such as duty-free shops, department stores, and local markets, tourists can systematically observe product assortments, market competitiveness, and the consumption patterns of local consumers. This process transcends mere information exposure and facilitates the accumulation of contextualized, experience-based knowledge.

Consequently, tourism-derived information can be conceptualized as experience-based information acquired through tourists’ observational, information-seeking, and interactive experiences during their visits. Unlike destination familiarity, a construct widely examined in the tourism marketing literature that reflects consumers’ overall knowledge and impressions of a destination (C. Chen & Lin, 2012), and country-of-origin effects, which refer to consumers’ indirect inferences about product quality based on a country’s image (Ortega-Egea & García-de-Frutos, 2021), tourism-derived information is formed through direct exposure to products within the authentic marketplace context of the destination country and provides consumers with specific evaluative cues for product evaluation during the pre-purchase stage. By offering direct and context-rich product information, tourism-derived information enables consumers to form more informed product evaluations and reduces perceived uncertainty regarding product quality and suitability.

## 3. Hypotheses Development

### 3.1. Tourism-Derived Information and Product Uncertainty

Product uncertainty refers to the degree to which consumers encounter challenges in assessing product attributes and forecasting future performance and suitability (Hong & Pavlou, 2014). In the context of CBEC, there exists a relatively high level of information asymmetry between sellers and consumers (Lee & Xiong, 2026), compelling consumers to depend on limited online information to assess product quality (Y. Guo et al., 2018). These limitations are primarily attributed to the structural characteristics of the online retail environment, in which products can only be presented through digital interfaces, making it difficult for sellers to effectively convey the actual quality and performance of their products (Gallino & Moreno, 2018; Mou et al., 2020). As a result, consumers may question the accuracy and completeness of the information provided, thereby increasing uncertainty in the product evaluation process (Dimoka et al., 2012). Moreover, differences in quality standards and product presentation formats across countries further impede consumers’ ability to evaluate and compare products using their prior knowledge and experience (Y. Guo et al., 2018).

In this context, product uncertainty is closely related to the extent to which consumers can clearly predict product quality and suitability (Hong & Pavlou, 2014). Prior research suggests that vivid and concrete information reduces consumers’ cognitive burden and enhances their understanding of product performance, thereby improving the predictability of outcomes (Flavián et al., 2017). Specifically, the greater the richness and realism of information, the more effectively consumers can evaluate products, ultimately leading to a reduction in uncertainty. This relationship has also been empirically supported in technology-mediated environments such as AR and VR (Daugherty et al., 2008; Sun et al., 2022; Wedel et al., 2020).

From this perspective, tourism experiences can serve as rich information sources that provide a higher level of vividness and contextual richness than virtual environments. Tourists directly observe and experience the local market environment in which products are produced, distributed, and consumed, thereby enabling them to accumulate diverse contextual information related to the products (Yim et al., 2007). Such experiences extend beyond mere information acquisition and enable consumers to form realistic judgments about how products are actually used and evaluated. This, in turn, enhances the predictability of product quality.

Furthermore, this relationship can be explained by the associative network theory. According to this theory, consumers’ memories are structured as a network of interconnected information nodes. When a specific piece of information is activated, related information is concurrently activated (Mandler, 1978). Destination-related memories formed through tourism experiences are linked to product-related information, and when consumers subsequently encounter these products, the associated experiences are activated simultaneously (De Nisco et al., 2017; Elliot et al., 2011). This cognitive activation facilitates the interpretation and evaluation of products originating from the destination country, thereby reducing product uncertainty. Accordingly, we propose the following hypothesis:

**H1.**  *Tourism-derived information negatively influences product uncertainty.*

### 3.2. Tourism-Derived Information and Product Familiarity

Product familiarity is a significant concept in consumer decision-making, referring to the extent of accumulated knowledge and experience related to a product (Baker et al., 1986). From an information-processing perspective, familiarity reflects not only the amount of knowledge but also the degree to which such knowledge is systematically organized in memory and how easily it can be accessed (Johnson & Russo, 1984). In other words, familiarity resides not merely in the accumulation of experience itself but in the cognitive accessibility formed through such experience.

Product familiarity can be classified into objective and subjective familiarity (C. W. Park & Lessig, 1981). Objective familiarity refers to the actual level of knowledge and experience that consumers possess regarding a product, whereas subjective familiarity reflects the degree to which consumers perceive themselves as knowledgeable and familiar with the product. Given that this study focuses on how information exposure and experience influence consumers’ perceptions, it emphasizes subjective familiarity.

Product familiarity can be developed not only through direct usage experience but also through indirect experiences, such as advertising and online reviews (Nepomuceno et al., 2014; C. W. Park & Lessig, 1981). In tourism research, this concept has been extensively explored in relation to destination familiarity. Previous research has indicated that tourists develop familiarity with a destination through both direct visitation experiences and exposure to information (Baloglu, 2001; C. Chen & Lin, 2012; Tan & Wu, 2016).

Extending this logic to the product level, tourism experiences facilitate the accumulation of memories and knowledge related to products originating from the destination country (Mainolfi & Marino, 2020). Tourists repeatedly observe and explore products in local markets, and, in some cases, directly experience them, thereby remaining continuously exposed to product-related information (Yim et al., 2007). Such repeated exposure and information accumulation enhance cognitive accessibility to products, leading consumers to perceive them as more familiar. Therefore, tourism-derived information is expected to serve as an important antecedent of product familiarity. Accordingly, the following hypothesis is proposed:

**H2.**  *Tourism-derived information positively influences product familiarity.*

### 3.3. Tourism-Derived Information and Perceived Diagnosticity

Perceived diagnosticity pertains to the degree to which information is perceived as useful for evaluating products and making informed decisions, serving as an effective basis for assessing product quality and performance (Jiang & Benbasat, 2004; Kempf & Smith, 1998).

Previous research has demonstrated that when consumers engage in direct interactions with a product or are exposed to detailed and vivid information, such information is more likely to be perceived as a valuable cue for product evaluation (Kempf & Smith, 1998). This pattern has been consistently observed in digital environments. For instance, Jiang and Benbasat (2004) identified that virtual experiences enabling consumers to explore product features or manipulate product images enhance product comprehension and strengthen perceived diagnosticity. Similarly, Uhm et al. (2022) demonstrated that augmented reality–based shopping environments enhance perceived diagnosticity by providing more vivid and concrete product information compared to traditional web-based environments. These findings imply that consumers are more likely to perceive information characterized by heightened interactivity and vividness as useful.

Consumers rely on various marketplace cues to infer product quality and value when direct evaluation is not feasible (Kirmani & Rao, 2000). Although digitally mediated information cues can partially reduce uncertainty in consumer decision-making, such environments remain inherently unable to convey the full sensory and contextual richness of products because consumers cannot physically handle or experience products in their actual usage context (Weathers et al., 2007). In contrast, tourism-derived information is acquired directly within the marketplace context in which products are actually marketed, displayed, and consumed. This enables consumers to verify product claims, compare alternatives, and evaluate product attributes using multiple sensory and contextual cues (Yim et al., 2007). As a result, consumers are more likely to perceive such information as a credible and useful basis for evaluating product quality and performance, thereby enhancing perceived diagnosticity. Accordingly, we propose the following hypothesis:

**H3.**  *Tourism-derived information positively influences perceived diagnosticity.*

### 3.4. Product Familiarity and Product Uncertainty

In the evaluation of products, consumers draw upon both externally provided information and the knowledge and experiences stored in their memories to interpret and assess products (Hong & Pavlou, 2014). This internal knowledge enhances the efficiency of information processing and diminishes the cognitive demands of product evaluation.

Product familiarity emerges as a critical factor influencing the manner in which consumers process information (Johnson & Russo, 1984). It is cultivated through the accumulation of prior experiences and knowledge, which enhances the accessibility and interpretability of product-related information (Coupey et al., 1998; Johnson & Russo, 1984). Previous research has indicated that consumers are more inclined to evaluate familiar products or brands positively and exhibit greater confidence in their purchase decisions (Mao & Lyu, 2017; Söderlund, 2002; Yunpeng & Khan, 2023). This is attributed to the fact that familiarity facilitates the retrieval and interpretation of product-related information, thereby reducing cognitive burden and enhancing clarity in the evaluation process (Gursoy, 2019; Hong & Pavlou, 2014).

Conversely, individuals with low product familiarity lack sufficient knowledge and experience, leading them to rely more heavily on external cues (Gursoy, 2019). These consumers, in particular, encounter challenges in accurately evaluating product quality or determining the extent to which products meet their needs (Dahl et al., 2001; Hong & Pavlou, 2014), which diminishes the predictability of product outcomes and heightens uncertainty. Therefore, product familiarity is expected to enhance information-processing efficiency and the clarity of judgment, thereby increasing the predictability of product quality and performance and ultimately reducing product uncertainty. Accordingly, we propose the following hypothesis:

**H4.**  *Product familiarity negatively influences consumers’ perceived product uncertainty.*

### 3.5. Perceived Diagnosticity and Product Uncertainty

During product evaluation, both the quantity and usefulness of information significantly impact consumers’ perceptions of uncertainty. Even when the same information is provided, evaluation outcomes may vary based on the degree to which consumers perceive the information as diagnostic (Jiang & Benbasat, 2004; Kempf & Smith, 1998; Pavlou et al., 2007). When information is perceived as diagnostic, consumers can understand product attributes more clearly and make more concrete judgments regarding product quality and performance (Jiang & Benbasat, 2004). Conversely, when information is perceived as non-diagnostic, consumers may still experience difficulties in evaluating the product, even if a sufficient amount of information is available, thereby maintaining a high level of uncertainty.

Empirical studies have shown that higher perceived diagnosticity significantly reduces uncertainty regarding product quality and fit (D. Liu & Yu, 2024; Pavlou et al., 2007; Sun et al., 2022; Uhm et al., 2022; Yan & Gong, 2023). Particularly in online shopping environments, even when a substantial amount of information is provided, if such information is not perceived as useful for product evaluation, it may instead increase cognitive confusion and elevate uncertainty (D. Liu & Yu, 2024; Sun et al., 2022; Yan & Gong, 2023). This suggests that in reducing uncertainty, the perceived usefulness of information is more important than the mere quantity of information.

When perceived diagnosticity is high, consumers perceive product-related information as directly applicable to their decision-making (Jiang & Benbasat, 2004). This enhances clarity in understanding product attributes and enables consumers to form more concrete expectations regarding product performance, thereby reducing ambiguity and ultimately lowering product uncertainty. Therefore, perceived diagnosticity is expected to be an important mechanism for alleviating product uncertainty. Accordingly, we propose the following hypothesis:

**H5.**  *Perceived diagnosticity negatively influences consumers’ perceived product uncertainty.*

### 3.6. Product Uncertainty and Cross-Border Purchase Intention

Purchase intention is defined as the likelihood or psychological tendency of a consumer to acquire a product (Lee & Xiong, 2026; Ma et al., 2025; Pavlou et al., 2007). In online shopping contexts, consumers cannot physically evaluate or interact with products prior to purchase. This inherent limitation generates significant uncertainty regarding product quality and performance (Ma et al., 2025; Pavlou et al., 2007; Sun et al., 2022). Under conditions of high uncertainty, consumers tend to focus more on potential losses and frequently overestimate the likelihood of unfavorable outcomes, even when their actual probability is low (Kahneman & Tversky, 2013). As uncertainty increases, consumers experience greater difficulty in predicting transaction outcomes and evaluating product quality, which contributes to the formation of perceived risk, defined as consumers’ subjective assessment of potential losses (Chiles & McMackin, 1996). Such risk perceptions weaken exchange relationships (Rousseau et al., 1998) and have been identified as significant inhibitors of purchase behavior in e-commerce settings (J. Park et al., 2005).

In the CBEC context, product uncertainty may be perceived as even greater due to variations in quality standards across countries, limited product information, and insufficient information about sellers (Lee & Xiong, 2026). From the perspective of transaction cost theory, uncertainty increases transaction costs by requiring consumers to expend additional time and effort on information search and product evaluation to assess product quality and suitability. As these costs increase, consumers become less confident in their purchase decisions and more reluctant to engage in transactions with uncertain outcomes (Williamson, 2010). Previous research has consistently demonstrated that product uncertainty is a significant factor that diminishes consumers’ purchase intention in CBEC environments (J. Guo et al., 2021; Huang & Chang, 2019; Mou et al., 2020; Wang et al., 2023). Accordingly, we propose the following hypothesis:

**H6.**  *Product uncertainty negatively influences cross-border purchase intention.*

### 3.7. The Moderating Role of Transaction Uncertainty

Consumers’ perceived uncertainty arises not only from the evaluation of product quality and performance (i.e., product uncertainty) but also from the transaction execution process in the CBEC context (Mou et al., 2020). Transaction uncertainty refers to consumers’ perceptions of the various risks and uncertainties that may arise during the execution of a transaction after a purchase contract has been established. This includes various uncertainties related to transaction execution, such as delivery problems, unexpected additional costs, concerns about personal information security, constraints in customs procedures, and difficulties in communicating with sellers (X. Chen & Kim, 2021; Huang & Chang, 2019; Mou et al., 2020).

Previous research has consistently demonstrated that transaction uncertainty is a significant factor that diminishes consumers’ purchase intentions (Forsythe et al., 2006; Kamalul Ariffin et al., 2018). In the CBEC context, consumers typically have relatively low levels of control and limited information regarding transaction processes, which increases perceived risk (K. Liu et al., 2025; Mou et al., 2020; Wagner et al., 2016).

This study underscores the distinction between product and transaction uncertainty, which manifest at different stages (Mou et al., 2020). Information obtained through tourism primarily mitigates uncertainty during the product evaluation phase. Nevertheless, even when consumers develop favorable evaluations of a product based on adequate information, they may still postpone or refrain from making purchase decisions if they perceive substantial uncertainty in the transaction execution process. Thus, transaction uncertainty functions as a boundary condition that influences the translation of product uncertainty into purchase intention in the CBEC context. Specifically, when transaction uncertainty is low, consumers’ confidence in a product is more likely to translate directly into purchase intention. When transaction uncertainty is high, even positive product evaluations may not culminate in actual purchase intention due to concerns about the transaction process. Therefore, transaction uncertainty is expected to moderate the association between product uncertainty and purchase intention. Accordingly, we propose the following hypothesis:

**H7.**  *Transaction uncertainty moderates the relationship between product uncertainty and cross-border purchase intention. In particular, the negative effect of product uncertainty on purchase intention is stronger when perceived transaction uncertainty is higher.*

**Figure 1:**
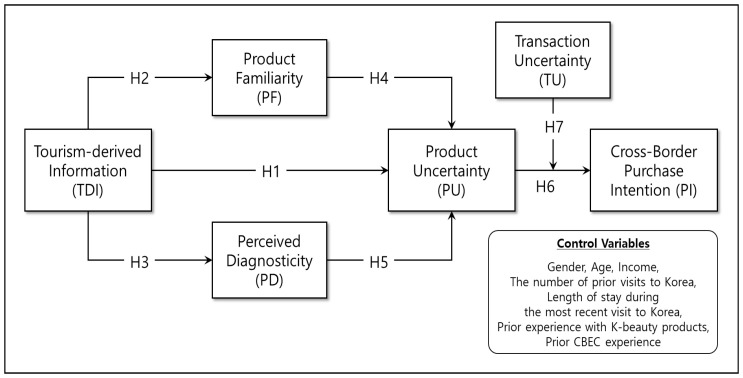
Research Model.

Figure 1 presents the proposed research model of this study. Drawing on transaction cost theory and uncertainty reduction theory, tourism-derived information is proposed to enhance product familiarity (H2) and perceived diagnosticity (H3), which in turn reduce product uncertainty (H4, H5), ultimately reducing cross-border purchase intention (H6). Tourism-derived information is also proposed to directly reduce product uncertainty (H1). Transaction uncertainty is introduced as a moderating variable that strengthens the negative relationship between product uncertainty and purchase intention (H7).

## 4. Methodology

### 4.1. Sample and Data

This study targeted Chinese consumers who had visited Korea within the past year and had encountered or purchased Korean cosmetics and beauty products (hereinafter referred to as K-beauty products) during their visit. The decision to limit the research scope to a single country and product category was made to minimize the potential confounding effects arising from heterogeneity in institutional environments, cultural contexts, and product characteristics, thereby enhancing the consistency and accuracy of measurement. If the research scope is not clearly specified, respondents may refer to different products or contexts when answering the survey, leading to heterogeneous evaluation criteria and increased measurement error.

China represents one of the largest CBEC markets globally (Ma et al., 2025), a major trading partner of Korea’s CBEC (KOTRA, 2025), and a key source market for inbound tourism in Korea (Korea Tourism Datalab, 2026). In this context, the China–Korea setting provides a particularly relevant empirical context for examining how tourism experiences translate into post-visit CBEC purchase behavior. This study specifically focuses on K-beauty products. These products have gained high recognition among international consumers due to the spread of the Korean Wave and the growing popularity of K-beauty (Global Cosmetics News, 2025). Moreover, cosmetic products are characterized by strong experiential attributes, as consumers often rely on direct sensory evaluation and usage-related judgments, such as texture, effectiveness, and skin suitability (Lian & Yen, 2013). Because of these characteristics, consumers find it difficult to fully assess product quality based solely on online information, which increases product uncertainty. Therefore, K-beauty products provide an appropriate setting for investigating how tourism-derived information shapes product familiarity, perceived diagnosticity, and product uncertainty.

To test the proposed hypotheses, this study conducted a questionnaire survey to collect data. The survey was administered through Wenjuanxing, a widely used online survey platform in China. To enhance sample diversity and minimize single-platform bias, the survey link was distributed through WeChat, Xiaohongshu, and online travel communities. The study employed a non-probability sampling method, specifically utilizing purposive sampling to select respondents based on predefined criteria. Two screening questions were employed to ensure respondent eligibility: whether the respondent had visited Korea within the past year and whether the respondent had encountered or purchased K-beauty products during the most recent visit. Only respondents who answered “yes” to both questions were included in this study. No monetary incentives were provided for participation. Nevertheless, because participation was voluntary, the possibility of self-selection bias cannot be completely ruled out. The survey was conducted over four weeks in March 2026. A total of 350 questionnaires were collected. After excluding responses that contained inconsistent answers or exhibited insincere response patterns, 325 valid questionnaires were retained for the final analysis.

Of the 325 valid survey responses, females accounted for 57.2% of the respondents, while males comprised 42.8%. Regarding age, the largest proportion of respondents was in their 20s (35.4%), followed by those in their 30s (28.3%) and 40s (22.2%), indicating that the sample was largely composed of young to middle-aged adults. Regarding education, a majority of the respondents held a bachelor’s degree (53.8%), followed by those with junior college degrees (31.4%), reflecting a relatively high level of educational attainment among participants. Regarding occupation, the largest group was company employees (57.5%), followed by self-employed individuals (13.2%) and public servants (11.1%). Regarding income, the largest proportions reported earnings of RMB 5000–6999 (31.7%) and RMB 7000 or above (33.2%). In addition, 68.3% of the respondents reported having prior experience with CBEC. Regarding prior K-beauty product experience, 25.8% of respondents reported having experience with K-beauty products prior to their most recent visit to Korea. In terms of the number of prior visits to Korea, 33.2% had visited twice, followed by three times (28.3%). The most frequently reported length of stay was 3–4 nights (34.8%). Detailed demographic information is summarized in Table 1.

### 4.2. Measurements

The measurement items employed in this study were derived from the existing literature and subsequently adjusted to align with the specific research context (see Appendix A). All constructs were measured using a five-point Likert scale, with response options ranging from 1 (“strongly disagree”) to 5 (“strongly agree”).

Tourism-derived information was measured using four items adapted from Cole and Scott (2004) and Oh et al. (2007). This refers to the degree to which consumers acquire information about K-beauty products through their visits to Korea. This construct was developed based on the concept of learning experience in tourism research, which refers to the degree to which tourists gain new knowledge through travel experiences (Ali et al., 2016; Cole & Scott, 2004; Oh et al., 2007; Suhartanto et al., 2020). Accordingly, the original items were modified to reflect information acquisition related to K-beauty products during a visit to Korea.

Perceived diagnosticity was assessed using four items adapted from Kempf and Smith (1998) and Jiang and Benbasat (2004). It pertains to the degree to which consumers regard tourism-derived information as beneficial for assessing K-beauty products.

Product familiarity was measured using four items adapted from Mao and Lyu (2017) and Söderlund (2002), which reflected consumers’ subjective perceptions of their knowledge and familiarity with K-beauty products. The two negatively worded items (PF2 and PF4) were reverse-coded prior to the analyses to ensure that higher scores consistently reflected higher levels of product familiarity.

Product uncertainty was assessed using four items adapted from Dimoka et al. (2012) and Hong and Pavlou (2014). It captures the extent to which consumers have difficulty in predicting the quality, performance, and fit of K-beauty products. Transaction uncertainty was measured using six items adapted from Huang and Chang (2019), Mou et al. (2020), and Wagner et al. (2016). This construct captures consumers’ concerns regarding potential problems in the CBEC transaction process, including delivery, additional costs, privacy, communication, returns, and customs clearance.

Cross-border purchase intention was assessed using four items adapted from Huang and Chang (2019) and Wagner et al. (2016). These items capture consumers’ likelihood and willingness to purchase K-beauty products through foreign-based CBEC platforms (e.g., Gmarket Global, Amazon). These platforms differ from domestically operated e-commerce platforms in China, such as Tmall Global and JD Worldwide, where consumers can purchase both domestic and imported products within a single integrated system (Lee et al., 2026). In contrast, foreign-based CBEC platforms are independently operated and typically require consumers to engage in separate cross-border payments, logistics, and customer service processes.

The survey also included sociodemographic variables, including gender, age, education, occupation, and income. In addition, gender, age, average monthly income, the number of prior visits to Korea, length of stay during the most recent visit to Korea, prior experience with K-beauty products before the most recent visit to Korea, and prior CBEC experience were included as control variables because these factors may influence information acquisition, product perceptions, and CBEC purchase decisions (Mao & Lyu, 2017; Mou et al., 2020; Yan & Gong, 2023).

Before the final survey was administered, three professors familiar with the research topic conducted a review of the questionnaire to ensure its content validity, and minor revisions were made accordingly. A pilot study was subsequently conducted with 50 eligible participants to assess the clarity of wording, scale reliability and validity, logical flow, and contextual appropriateness. Cronbach’s alpha values ranged from 0.825 to 0.898, while factor loadings varied from 0.675 to 0.852, demonstrating an acceptable level of reliability and validity for exploratory research (Nunnally & Bernstein, 1995). The wording of the questionnaires was slightly modified based on respondents’ feedback.

## 5. Results

This study utilized SmartPLS 4.0 to perform partial least squares structural equation modeling (PLS-SEM). PLS-SEM is considered particularly appropriate for examining complex research models in exploratory settings. Unlike covariance-based SEM (CB-SEM), which primarily focuses on theory confirmation and overall model fit assessment, PLS-SEM is well-suited for prediction-oriented research aimed at explaining variance in key endogenous constructs (Hair et al., 2019). Given that the primary objective of this study is to explain and predict consumers’ cross-border purchase intention through a series of mediating and moderating relationships, PLS-SEM was considered the more appropriate analytical method for estimating complex causal paths. In this study, all constructs were modeled reflectively. Following the two-step procedure recommended in the literature, the measurement model was first evaluated to assess reliability and validity; subsequently, the structural model was analyzed to examine the hypothesized relationships.

### 5.1. Measurement Model

To evaluate the measurement model, this study assessed reliability, convergent validity, and discriminant validity in accordance with established guidelines. First, internal consistency reliability was assessed through Cronbach’s alpha (CA) and composite reliability (CR). As presented in Table 2, the CA values ranged from 0.857 to 0.916, and the CR values ranged from 0.858 to 0.917, both exceeding the recommended threshold of 0.70 (Fornell & Larcker, 1981). These results demonstrate adequate internal consistency across all constructs.

Second, convergent validity was assessed by evaluating factor loadings and average variance extracted (AVE). As presented in Table 2, all factor loadings exceeded 0.70, and AVE values ranged from 0.699 to 0.738, surpassing the recommended threshold of 0.50 (Fornell & Larcker, 1981). These results confirm that all constructs demonstrate sufficient convergent validity.

Third, discriminant validity was evaluated using the heterotrait–monotrait ratio (HTMT) criterion (Henseler et al., 2015). As shown in Table 3, all HTMT values were below the conservative threshold of 0.85, thereby confirming the establishment of discriminant validity for all constructs.

Finally, multicollinearity was evaluated through the use of the variance inflation factor (VIF) values. As presented in Table 2, the maximum VIF value was 2.917, which is considerably below the recommended threshold of 10, suggesting that multicollinearity is not a concern in this study (Hair et al., 2010).

### 5.2. Common Method Bias

Considering that the data were obtained from a single source using self-reported measures, common method bias (CMB) may pose a concern. To address this issue, both procedural and statistical remedies were applied. Procedurally, respondents were guaranteed anonymity and confidentiality and informed that no correct or incorrect answers existed, thereby alleviating evaluation apprehension and social desirability bias. Additionally, different scale formats were used for the key constructs and control variables to minimize response pattern bias. Statistically, Harman’s single-factor test was performed. The findings revealed that the first factor explained 16.229% of the total variance, which is below the critical threshold of 50% (Podsakoff et al., 2003), suggesting that common method bias is unlikely to be a serious concern. In addition, all variance inflation factor (VIF) values ranged from 1.000 to 1.333, which is well below the recommended threshold of 3.3 (Kock, 2015). This finding provides further evidence that common method bias is unlikely to threaten the validity of the results.

### 5.3. Structural Model Results

**Figure 2:**
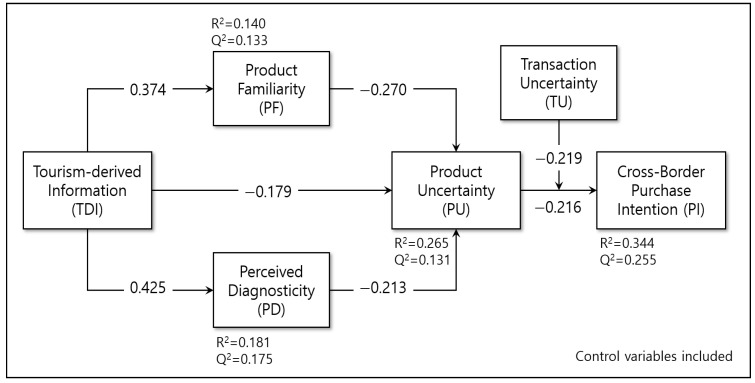
Results of Structural Assessment.

To examine the proposed research model and hypotheses, PLS-SEM was conducted using SmartPLS 4.0 with bootstrapping (5000 resamples) (Hair et al., 2019). The results of the structural model, including the path coefficients, t-values, significance levels (*p*-values), and determination coefficients (R^2^), are presented in Figure 2 and Table 4. The results show that the R^2^ values for product familiarity, perceived diagnosticity, product uncertainty, and purchase intention were 0.140, 0.181, 0.265, and 0.344, respectively. Moreover, we also applied Q^2^ to evaluate the cross-validated redundancy of the structural model. The Q^2^ values of product familiarity, perceived diagnosticity, product uncertainty, and purchase intention were 0.133, 0.175, 0.131, and 0.255, respectively. As all Q^2^ values were greater than zero, the model exhibited satisfactory predictive relevance (Hair et al., 2019).

As shown in Table 4, tourism-derived information had a significant negative effect on product uncertainty (β = −0.179, *p* < 0.05), thereby supporting H1. In addition, tourism-derived information positively influenced both product familiarity (β = 0.374, *p* < 0.001) and perceived diagnosticity (β = 0.425, *p* < 0.001), supporting H2 and H3. Regarding the antecedents of product uncertainty, both product familiarity (β = −0.270, *p* < 0.001) and perceived diagnosticity (β = −0.213, *p* < 0.001) significantly reduced product uncertainty, supporting H4 and H5. To further examine the underlying mechanisms, indirect effects were assessed using bootstrapping procedures. The results indicated that product familiarity (β = −0.101, *p* < 0.001) and perceived diagnosticity (β = −0.091, *p* < 0.05) significantly mediated the relationship between tourism-derived information and product uncertainty.

Furthermore, product uncertainty had a significant negative effect on cross-border purchase intention (β = −0.216, *p* < 0.001), supporting H6. Indirect effect analysis further revealed that product uncertainty served as a key mediating mechanism linking tourism-derived information to cross-border purchase intention. Specifically, the indirect effect of tourism-derived information on purchase intention via product uncertainty (β = 0.039, *p* < 0.05) was significant. In addition, both product familiarity (β = 0.058, *p* < 0.05) and perceived diagnosticity (β = 0.046, *p* < 0.05) had significant indirect effects on cross-border purchase intention through product uncertainty. Moreover, the sequential indirect effects were significant. The pathways from tourism-derived information to product familiarity, product uncertainty, and cross-border purchase intention (β = 0.022, *p* < 0.05) and from tourism-derived information to perceived diagnosticity, product uncertainty, and cross-border purchase intention (β = 0.020, *p* < 0.05) were supported.

**Figure 3:**
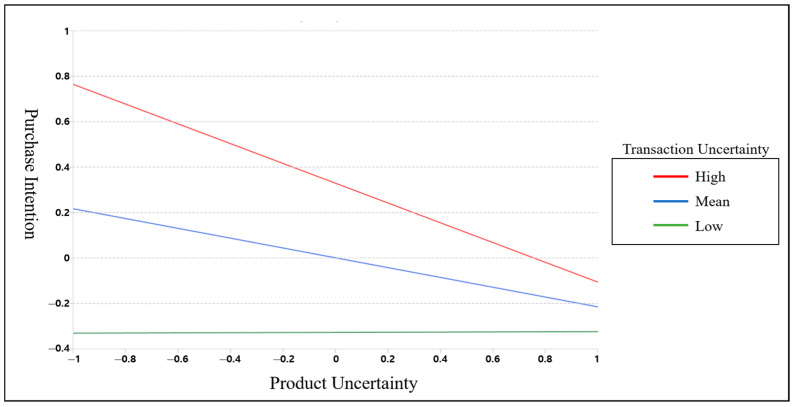
Simple Slope Diagram of the Moderating Effect of Transaction Uncertainty.

Finally, transaction uncertainty had a significant negative effect on cross-border purchase intention (β = −0.329, *p* < 0.001). In addition, the interaction between product uncertainty and transaction uncertainty on cross-border purchase intention was significant (β = −0.219, *p* < 0.001), supporting H7. The simple slope analysis (M ± 1 SD), as depicted in Figure 3, further indicated that the negative relationship between product uncertainty and cross-border purchase intention became stronger as transaction uncertainty increased. Specifically, the effect of product uncertainty was strongest under high transaction uncertainty and was significantly attenuated under low transaction uncertainty.

## 6. Discussion and Implications

### 6.1. Discussion

This study aimed to explore the impact of tourism-derived information on consumers’ purchase intention in the CBEC context and to empirically examine the cognitive mechanisms and boundary conditions that underpin this process. The findings reveal that tourism-derived information significantly enhances both product familiarity and perceived diagnosticity, while also directly mitigating product uncertainty. Moreover, product familiarity and perceived diagnosticity were identified as critical determinants in reducing product uncertainty, which, in turn, negatively influences cross-border purchase intention. Furthermore, transaction uncertainty was found to act as an important boundary condition that strengthens the relationship between product uncertainty and purchase intention.

First, the findings demonstrate that tourism-derived information serves as a vital source for reducing product uncertainty in the CBEC context. Tourism experiences extend beyond mere leisure activities, providing experience-based information that can be leveraged in subsequent CBEC contexts. During tourism, consumers do not encounter products in a fragmented manner; rather, they observe, compare, and occasionally directly experience products within the country-of-origin market context, thereby accumulating more concrete and realistic information about them. Such information enables consumers to evaluate products more clearly, ultimately reducing uncertainty in product evaluation.

Second, this study confirms that product familiarity and perceived diagnosticity function as key cognitive mechanisms through which tourism-derived information influences product uncertainty. Repeated exposure and direct experience in the country-of-origin market facilitate the accumulation of product-related knowledge, leading consumers to perceive products as more familiar. Simultaneously, such experience-based information is perceived as useful for product evaluation, thereby enhancing perceived diagnosticity. Furthermore, the finding that both product familiarity and perceived diagnosticity significantly reduce product uncertainty is noteworthy. These results suggest that reducing uncertainty depends not merely on exposure to information but also on how such information is cognitively processed and interpreted. Although tourism-derived information significantly influenced product familiarity and perceived diagnosticity, its explanatory power was relatively limited. This suggests that product familiarity and perceived diagnosticity not shaped not solely by tourism experiences but also by a variety of other information sources. Nevertheless, the findings indicate that tourism-derived information uniquely contributes to product evaluation-related cognitive judgments and serves as an important informational resource that complements existing digital information sources in the CBEC context.

Third, product uncertainty was found to be a key factor that inhibits cross-border purchase intention. This finding is consistent with prior research, which suggests that when consumers perceive greater difficulty in predicting product quality or performance, they are more inclined to postpone or avoid purchase decisions (Mou et al., 2020; Wang et al., 2023; L. Zhu et al., 2020). In the CBEC context, such uncertainty may be exacerbated by information asymmetry and institutional differences, reaffirming that product uncertainty remains a critical constraint on consumer purchase intention.

Fourth, transaction uncertainty was found not only to moderate the relationship between product uncertainty and purchase intention but also to exert a significant direct effect on purchase intention. This finding suggests that consumer decision-making in the CBEC context is influenced not only by product evaluations but also by perceptions of the transaction process itself. Even when consumers hold favorable evaluations of a product, they may still hesitate to proceed with the purchase if they perceive risks associated with the transaction process, such as payment security, international shipping, or customs procedures. Notably, the direct effect of transaction uncertainty on purchase intention was stronger than that of product uncertainty. This suggests that concerns about the transaction process may exert a greater inhibitory influence on purchase decisions than concerns about the product itself in the CBEC context. These findings imply that reducing product uncertainty alone may be insufficient to stimulate cross-border purchase intention unless transaction-related uncertainties are adequately addressed.

Finally, the indirect effect analysis indicates that tourism experience does not directly influence cross-border purchase intention; rather, it affects purchase intention through cognitive pathways in which product familiarity and perceived diagnosticity reduce product uncertainty. In other words, tourism experience enables consumers to better understand and evaluate products, thereby reducing uncertainty and ultimately enhancing their intention to purchase cross-border products. While digital technologies such as AR and VR are increasingly adopted by platforms to reduce product uncertainty, the findings of this study suggest that consumers’ offline experiential knowledge functions as an equally important cognitive resource in the CBEC purchase decision-making process.

### 6.2. Theoretical Implications

This study provides several theoretical contributions by integrating insights from tourism, trade, and CBEC research. First, it extends prior research that explained the relationship between tourism and international trade at the macro-level to the micro-level consumer decision-making process. While previous studies have primarily focused on the relationship between tourist inflows and international trade outcomes (Brau & Maria Pinna, 2013; El-Sahli, 2018; Santana-Gallego et al., 2016), they have provided limited explanations of how tourism experience translates into actual purchase behavior. This study empirically identifies the process through which tourism-derived information reduces product uncertainty via cognitive mechanisms—specifically, product familiarity and perceived diagnosticity—ultimately leading to cross-border purchase intention. In doing so, it provides a more refined explanation of the micro-level pathway through which tourism experience is translated into consumer behavior on e-commerce platforms post-travel.

Second, this study reconceptualizes tourism experience not merely as a determinant of destination-related attitudes but as an experience-based informational resource that is actively utilized in post-visit purchase decision-making. Previous research has primarily focused on the effects of tourism experiences on destination image formation and product preferences (De Nisco et al., 2017; Mainolfi & Marino, 2020; Tan & Wu, 2016), while existing CBEC studies have largely emphasized platform-mediated mechanisms for reducing uncertainty, such as online reviews, seller reputation, and digital technologies (Lu & Chen, 2021; Sun et al., 2022; Xu et al., 2024; W. Zhu et al., 2019). In contrast, this study demonstrates that information acquired through tourism experiences serves as a cognitive basis for consumers’ interpretation and evaluation of products in the CBEC context. The findings further suggest that offline experiential knowledge can serve as an uncertainty-reducing resource that complements existing digital information sources, offering a new perspective that tourism and e-commerce, which have traditionally been treated as separate domains, can be meaningfully integrated through consumer information processing.

Third, this study conceptualizes uncertainty in the CBEC context as a multidimensional construct and empirically demonstrates its role. While many prior studies have treated uncertainty or risk as a unidimensional perception (Lee & Xiong, 2026; K. Liu et al., 2025; Wagner et al., 2016) or merely as predictors of purchase behavior (Mou et al., 2020), this study distinguishes between product uncertainty at the product evaluation stage and transaction uncertainty at the transaction execution stage. Furthermore, by empirically demonstrating that transaction uncertainty moderates the relationship between product uncertainty and purchase intention, this study highlights the need to understand consumer decision-making in CBEC as a structured and multistage process.

### 6.3. Practical Implications

Several practical implications for tourism marketers, CBEC platforms, international retailers, and policymakers can be derived from the findings of this study. First, tourism should be leveraged as a strategic pre-entry channel for supporting product exports. Tourism is not merely a service activity that induces on-site consumption; it also functions as a pre-purchase learning process that may translate into post-visit product purchases through online platforms. Accordingly, firms should view tourists not as one-time visitors but as potential long-term customers who can be converted into repeat buyers through CBEC. In particular, for products with strong experiential attributes, such as cosmetics, firms should provide opportunities for direct product experience through experiential retail spaces, flagship stores, and duty-free shops to establish a cognitive foundation for future online purchases.

Second, tourism marketers should develop strategies, in collaboration with international retailers, that facilitate product discovery and trial during the travel experience. Product demonstration zones, experiential retail spaces, destination-linked brand stores, and tourism–shopping integration programs can help tourists acquire firsthand product knowledge and reduce uncertainty regarding product quality and suitability. These initiatives may increase the likelihood that tourists continue to purchase destination-related products through CBEC platforms after returning home.

Third, international retailers and CBEC platforms should develop information integration strategies that connect tourism experiences with online purchasing environments. The findings indicate that consumers’ understanding of products and evaluation criteria formed during tourism plays a pivotal role in their post-visit purchase decisions. Therefore, product information, brand messaging, and usage instructions should be presented consistently across both tourism settings and online platforms, enabling consumers to evaluate products using a unified reference framework. Furthermore, international retailers should establish digital systems—such as QR code-based tools or dedicated mobile applications—that allow tourists to store product information acquired during their travels and seamlessly access it on CBEC platforms after returning home. In addition, CBEC platforms may utilize AI-based personalization systems to prioritize products associated with destinations previously visited by consumers or provide destination-based product collections, thereby more systematically converting offline tourism experiences into online purchases.

Fourth, international retailers and brand firms should develop strategies to maintain ongoing purchasing relationships with tourists after they return home. To this end, providing multilingual digital content, destination-linked membership programs, and personalized follow-up communications can help sustain the product familiarity and perceived diagnosticity developed during tourism. Such initiatives may ultimately translate these cognitive foundations into continued cross-border purchasing behavior.

Finally, reducing transaction uncertainty is crucial for the expansion of CBEC. Even when product uncertainty is minimized, high levels of perceived uncertainty in the transaction process may constrain purchase intention. Accordingly, platform operators, sellers, and policymakers should reduce transaction uncertainty by enhancing logistics reliability, strengthening payment security, improving price transparency, simplifying return procedures, streamlining customs processes, and providing multilingual customer support. Such efforts can create an environment in which tourism experiences are more effectively translated into sustained cross-border purchasing behavior.

### 6.4. Limitations

This study has several limitations that suggest avenues for future research. First, the reliance on cross-sectional data constrains the ability to rigorously establish causal relationships among the variables. Future research should employ longitudinal data or experimental methodologies to more precisely examine the process through which tourism experiences translate into CBEC purchasing behavior over time. Second, this study employed a non-probability purposive sampling approach and relied on voluntary participation. Although screening procedures were applied to ensure respondent eligibility, the possibility of self-selection bias cannot be completely ruled out. In particular, consumers with greater interest in Korean tourism, K-beauty products, or CBEC may have been more likely to participate in the survey. Future studies should employ probability-based sampling techniques or utilize platform-based behavioral data to improve sample representativeness and reduce potential selection bias. Third, the sample in this study was limited to Chinese consumers who had visited Korea and to a specific product category, namely K-beauty products, which may limit the external validity of the findings. The results may not be directly generalizable to other countries, tourism destinations, or product categories with varying levels of experiential attributes. Therefore, future studies should conduct cross-country comparative research and incorporate a broader range of tourism and product contexts to enhance the generalizability of the findings. Fourth, this study conceptualized tourism-derived information as a unidimensional construct. However, consumers acquire different types of information through various channels during tourism, including observation, direct experience, and interactions with local staff and other consumers. Future research should distinguish among different types of information and levels of experiential depth to provide a more nuanced understanding of how tourism-derived information influences consumer decision-making. Fifth, this study explained the cognitive mechanism primarily through product familiarity and perceived diagnosticity, without fully accounting for other psychological factors, such as trust, psychological distance, and emotional responses. Moreover, the relatively low explanatory power observed for product familiarity and perceived diagnosticity suggests that these cognitive evaluations are shaped not only by tourism-derived information but also by a variety of informational and psychological factors. Future research should incorporate additional antecedents and psychological mechanisms to develop a more comprehensive explanatory model. Finally, this study used cross-border purchase intention as the dependent variable and does not capture actual purchase behavior. Future research should use actual purchase data or platform-based behavioral data to further validate and extend the findings.

## Figures and Tables

**Table 1:** Demographic Information of the Respondents (N = 325).

Demographics	Category	Count	Rate (%)
Gender	Male	139	42.8
	Female	186	57.2
Age	Under 20 years old	28	8.6
	20–29 years old	115	35.4
	30–39 years old	92	28.3
	40–49 years old	72	22.2
	50 years old or above	18	5.5
Education	High school graduate or below	22	6.8
	Junior college graduate	102	31.4
	Bachelor’s degree	175	53.8
	Graduate school or above	26	8.0
Occupation	Company employee	187	57.5
	Public servant	36	11.1
	Professional/technical worker	26	8.0
	Self-employed	43	13.2
	Student	28	8.6
	Other	5	1.6
Average monthly income	Less than RMB 1000	32	9.8
	RMB 1000~2999	20	6.2
	RMB 3000~4999	62	19.1
	RMB 5000~6999	103	31.7
	RMB 7000 or above	108	33.2
Prior CBEC experience	Yes	222	68.3
	No	103	31.7
Prior K-beauty product experience	Yes	84	25.8
	No	241	74.2
The number of prior visits to Korea	1 time	71	21.9
	2 times	108	33.2
	3 times	92	28.3
	4 times or more	54	16.6
Length of stay (most recent visit to Korea)	2 nights or below	83	25.5
	3–4 nights	113	34.8
	5–6 nights	61	18.8
	7 nights or above	68	20.9

**Table 2:** Results of the Reliability, Validity, and Multicollinearity.

Construct	Items	Factor Loading	VIF	CA	CR	AVE
Tourism-derived information (TDI)	TDI1	0.846	2.043	0.860	0.861	0.704
	TDI2	0.836	1.956			
	TDI3	0.834	1.933			
	TDI4	0.841	2.066			
Product familiarity (PF)	PF1	0.841	1.986	0.870	0.870	0.719
	PF2	0.842	2.072			
	PF3	0.861	2.100			
	PF4	0.848	2.287			
Perceived diagnosticity (PD)	PD1	0.844	1.994	0.857	0.858	0.699
	PD2	0.815	1.861			
	PD3	0.839	2.048			
	PD4	0.847	2.083			
Product uncertainty (PU)	PU1	0.847	2.150	0.865	0.866	0.712
	PU2	0.845	2.053			
	PU3	0.858	2.214			
	PU4	0.826	1.878			
Transaction uncertainty (TU)	TU1	0.837	2.401	0.916	0.917	0.704
	TU2	0.830	2.517			
	TU3	0.815	2.156			
	TU4	0.837	2.377			
	TU5	0.842	2.444			
	TU6	0.870	2.917			
Cross-border purchase intention (PI)	PI1	0.863	2.325	0.882	0.882	0.738
	PI2	0.856	2.238			
	PI3	0.861	2.210			
	PI4	0.856	2.240			

**Table 3:** Heterotrait–Monotrait (HTMT) Ratio.

	TDI	PF	PD	PU	TU	PI
TDI						
PF	0.431					
PD	0.495	0.462				
PU	0.429	0.487	0.460			
TU	0.539	0.538	0.474	0.537		
PI	0.491	0.480	0.526	0.486	0.533	

Notes: TDI, tourism-derived information; PF, product familiarity; PD, perceived diagnosticity; PU, product uncertainty; TU, transaction uncertainty; PI, cross-border purchase intention.

**Table 4:** Summary of the Results.

	Pathway	Standardized Coefficient (β)	Standard Deviation	t Value	f^2^	Result
Hypotheses tests						
H1:	TDI → PU	−0.179	0.055	3.236 *	0.034	Supported
H2:	TDI → PF	0.374	0.047	7.910 **	0.163	Supported
H3:	TDI → PD	0.425	0.048	8.939 **	0.221	Supported
H4:	PF → PU	−0.270	0.052	5.236 **	0.079	Supported
H5:	PD → PU	−0.213	0.057	3.761 **	0.047	Supported
H6:	PU → PI	−0.216	0.054	3.962 **	0.053	Supported
H7:	PU × TU → PI	−0.219	0.047	4.659 **	0.068	Supported
Supplementary Analysis: Indirect effect						
TDI → PF → PU		−0.101	0.023	4.393 **		-
TDI → PD → PU		−0.091	0.027	3.417 *		-
TDI → PU → PI		0.039	0.016	2.411 *		-
PF → PU → PI		0.058	0.019	3.104 *		-
PD → PU → PI		0.046	0.019	2.467 *		-
TDI → PF → PU → PI		0.022	0.008	2.842 *		-
TDI → PD → PU → PI		0.020	0.008	2.340 *		-
Control Variables						
Gender → PI		−0.055	0.095	0.581	0.001	-
Age → PI		0.059	0.051	1.144	0.004	-
Income → PI		−0.068	0.053	1.285	0.005	-
Korea visits → PI		−0.018	0.048	0.372	0.000	-
Stay duration → PI		0.046	0.044	1.036	0.003	-
K-beauty experience → PI		−0.225	0.104	2.170 *	0.014	-
CBEC experience → PI		−0.050	0.094	0.534	0.001	-

Notes: 1. TDI, tourism-derived information; PF, product familiarity; PD, perceived diagnosticity; PU, product uncertainty; TU, transaction uncertainty; PI, cross-border purchase intention; Korea visits, the number of prior visits to Korea; Stay duration, length of stay during the most recent visit to Korea; K-beauty experience, prior experience with K-beauty products; CBEC experience, prior CBEC experience. 2. * *p* < 0.05, ** *p* < 0.001.

## Data Availability

The data presented in this study are available from the corresponding author upon reasonable request due to privacy and ethical restrictions approved by the Ethics Committee of Shandong Xiehe University.

## Appendix A. Measurement Items

Tourism-derived Information	
TDI1	Through my visit to Korea, I gained new knowledge about K-beauty products.
TDI2	My visit to Korea helped me learn more about K-beauty products.
TDI3	During my visit to Korea, I obtained useful information about K-beauty products.
TDI4	My visit to Korea was a meaningful experience that broadened my understanding of K-beauty products.
Product Familiarity	
PF1	I know a lot about K-beauty products.
PF2	I do not feel very knowledgeable about K-beauty products. (Reverse scored)
PF3	I feel familiar with K-beauty products.
PF4	Compared to other people, I know less about K-beauty products. (Reverse scored)
Perceived Diagnosticity	
The information I obtained about K-beauty products...	
PD1	helps me judge the quality and performance of K-beauty products.
PD2	is useful for accurately evaluating K-beauty products.
PD3	is useful for making an overall evaluation of K-beauty products.
PD4	helps me gain a more realistic understanding of K-beauty products.
Product Uncertainty	
When purchasing K-beauty products through cross-border e-commerce platforms...	
PU1	I am uncertain about the quality of K-beauty products.
PU2	I am uncertain about the future effectiveness of K-beauty products.
PU3	I am uncertain whether K-beauty products will meet my needs.
PU4	I am uncertain whether K-beauty products will match my preferences.
Transaction Uncertainty	
When purchasing K-beauty products through cross-border e-commerce platforms...	
TU1	I am concerned that delivery problems may occur.
TU2	I am concerned that unexpected additional costs (e.g., customs duties or extra fees) may arise.
TU3	I am concerned that my personal or payment information may be misused.
TU4	I am concerned that communication difficulties with sellers may occur.
TU5	I am concerned that returning products may be difficult.
TU6	I am concerned that customs-related problems may occur.
Cross-border Purchase Intention	
PI1	It is likely that I will purchase K-beauty products through cross-border e-commerce platforms (e.g., Gmarket Global, CJ OliveYoung Global) in the near future.
PI2	Given the opportunity, I intend to purchase K-beauty products through cross-border e-commerce platforms.
PI3	I would like to purchase K-beauty products through cross-border e-commerce platforms.
PI4	I plan to purchase K-beauty products through cross-border e-commerce platforms in the future.
